# Co‐infections and immune‐evading viral hybrids: A perspective

**DOI:** 10.1002/hsr2.1780

**Published:** 2024-01-03

**Authors:** Ryan Varghese, Dileep Kumar, Rohit Sharma, Shopnil Akash

**Affiliations:** ^1^ Department of Pharmaceutical Chemistry, Poona College of Pharmacy Bharati Vidyapeeth (Deemed to be) University Pune Maharashtra India; ^2^ Department of Clinical Pharmacology, Advanced Centre for Treatment, Research, and Education in Cancer Tata Memorial Centre, Kharghar Navi Mumbai India; ^3^ Department of Medical and Health Sciences Homi Bhabha National Institute Anushakti Nagar Mumbai Maharashtra India; ^4^ Department of Entomology University of California Davis California USA; ^5^ UC Davis Comprehensive Cancer Center University of California Davis California USA; ^6^ Department of Rasa Shastra and Bhaishajya Kalpana, Faculty of Ayurveda, Institute of Medical Sciences Banaras Hindu University Varanasi Uttar Pradesh India; ^7^ Department of Pharmacy, Faculty of Allied Health Science Daffodil International University Daffodil Smart City, Ashulia, Savar Dhaka Bangladesh

**Keywords:** co‐infection, immune evasion, influenza A virus, respiratory syncytial virus, respiratory viral infections, viral hybrid

## Abstract

**Background and Aims:**

Co‐infections occur when two or more different types of pathogens infect the same host at the same time. Initially, it may develop via a primary infection and then later segue into a superinfection. Although some research suggests that coinfections do not affect the effect of disease outcomes, alternate evidence says otherwise. While the disease outcomes are frequently influenced by the interactions between many viruses, how these viruses interact during coinfections is poorly understood. This article aims to shed light on the interaction between viruses at a cellular and subcellular level, and the clinical implications for the same.

**Methods:**

The articles were sought by conducting a thorough literature search on Google Scholar, ScienceDirect, PubMed, PubMed Central, Dimensions, and EBSCO Host, using keywords such as coinfections, virus, viral hybrids, and superinfection. The articles pertinent to the concept were then included.

**Results:**

There is a growing body of evidence that suggests the formation of hybrid viral particles (HVPs) which conjugate at the cellular and subcellular level. While the formation of HVPs is bizarre, it may potentially have a profound effect on the clinical manifestations.

**Conclusion:**

While there has been evidence of the formation of HVPs between a couple of viruses, researchers fear the existence of several other combinations, including zoonotic viruses. While this could be detrimental to the human race both at an individual—as well as a community‐level, an in‐depth understanding of the same may help in better management of the clinical manifestations of the disease.

Dear Editor,

Since the emergence of the COVID‐19 pandemic and the upcoming variants of the causative severe acute respiratory syndrome coronavirus‐2 (SARS‐CoV‐2) virus (such as BF.7, XBB.1.5, BQ.1, and BQ.1.1),^1^ health experts and researchers fear a “tridemic” outbreak that confluences fatalities from SARS‐CoV‐2, respiratory syncytial virus (RSV), and influenza.^2^ This has necessitated the close monitoring of viral outbreaks including monkeypox,^3^ tomato flu,^4^ and lumpy skin disease, to list a few.

The growth and multiplication of viruses majorly depend on the invasion of a narrow spectrum of cell types, in a property termed “tropism.” A similarity in tropism results in phylogenetically different viruses invading and thriving in cells of the same host, thereby leading to the coexistence of viral communities.^5^ Globally, respiratory viral infections (RVIs) are the leading cause of morbidity and mortality. The perils associated with RVIs have been exacerbated by viral co‐infection, as supported by numerous studies. Of all RVI cases, 10%–30% include co‐infections and are conspicuously observed in children.^6^ Notable viruses partaking in co‐infections include RSV, adenovirus, human rhinovirus, influenza A virus (IAV), and influenza B virus (IBV).^7^ While some studies suggest little or no overall change in the clinical outcome of RVIs following co‐infections,^8^, ^9^ others claim an increased incidence of viral pneumonia,^10^, ^11^ thereby leaving their effect on clinical manifestations unclear.^5^ The authors opine that this effect would majorly depend upon the tissue infected, the type of viruses involved, their relative counts, and their mutual interactions.

Although co‐infections are rather common, their responses when invading and replicating within a single cell remain ill‐defined. Haney et al. aimed to understand inter‐viral interactions during a co‐infection. The team used a mixed inoculum of RSV and IAV in a human lung‐derived cell line (A549), compared to single viruses inoculated as controls.^5^

The results reported extracellular and membrane‐associated filamentous structures that substantiate the formation of tree‐like hybrid viral particles (HVPs) that express ribonucleoproteins and surface glycoproteins of both RSV and IAV.^5^ Furthermore, these HVPs demonstrate a survival advantage as they employ the RSV fusion glycoprotein to bypass the neutralizing anti‐IAV antibodies to invade and replicate in cells devoid of IAV receptors.^5^ After the hybridization, IAV infects a broader spectrum and a relatively higher number of human cells, thereby increasing its potential to penetrate deeper into the respiratory tract and increase the severity of infections. However, the hybridization with IAV significantly impeded the replication capacity of RSV. Unlike the viral competition, such an incidence of viral co‐option for their own benefit has been a novel incidence.^5^

Reportedly, these HVPs could not only infect individual respiratory cells, but also the cultured layer of cells. As cells in human tissue are firmly arrayed, viruses, as well as HVPs, would need to enter and exit the cells in a particular manner. Since RSV tends to reach the lower pulmonary lobes, this hybridization could trigger serious and potentially fatal viral pneumonia or other similar lung infections.^12^ However, further in vivo and clinical testing would be recommended to substantiate the infectivity and severity of their combined infection.

Although the current body of evidence is limited to the hybridization of RSV and IAV, scientists believe that several combinations of viral hybrids exist, and animal viruses are no exception.^12^ As such hybridizations could be detrimental or fatal at both individual‐ and community‐level, it is of paramount importance to prevent incidences of co‐infection and/or superinfection. Nonetheless, there is a pressing need to research and elucidate the occurrence of co‐infections to obtain a panoramic view of the latter.

However, the counter‐view questions the infectivity potential of these hybrids as the study is in vitro and has not been clinically validated. Additionally, further research would be imperative to substantiate the probability and mechanism of the combined entry of these viruses within a single cell, in the real world. Furthermore, in the case of co‐infections, the competition between viruses only allows one virus to enter the cells, and subsequently replicate. Early pandemic research posits differences in the binding energies and efficiencies of different viruses to human cells, ultimately leading to competition.^2^

In conclusion, a deeper understanding of viral behavior in a co‐infection could aid in devising biological models and predicting the severity or the clinical outcome of the co‐infection. This would not only help to prevent these infections effectively but would also ensure better diagnosis and prognosis of these infections. Subsequently, newer fixed‐dose combinations and/or repurposing of preapproved drugs^3^ could be strategized to combat co‐infections prevalent in a particular geographical or socioeconomic sub‐stratum.

## AUTHOR CONTRIBUTIONS

**Ryan Varghese**: Conceptualization; data curation; formal analysis; investigation; validation; visualization; writing—original draft. **Dileep Kumar**: Conceptualization; data curation; formal analysis; resources; software; writing—original draft; writing—review and editing. **Rohit Sharma**: Resources; software; supervision; validation; visualization; writing—review and editing. **Shopnil Akash**: Data curation; validation; visualization; writing—review and editing. All authors have read and approved the final version of the manuscript. The Guarantor had full access to all of the data in this study and takes complete responsibility for the integrity of the data and the accuracy of the data analysis.

## CONFLICT OF INTEREST STATEMENT

The authors declare no conflict of interest.

## TRANSPARENCY STATEMENT

The lead author Dileep Kumar, Rohit Sharma, Shopnil Akash affirms that this manuscript is an honest, accurate, and transparent account of the study being reported; that no important aspects of the study have been omitted; and that any discrepancies from the study as planned (and, if relevant, registered) have been explained.

## Data Availability

The authors confirm that the data supporting the findings of this study are available within the article.
